# Dielectric Properties of Bi_2/3_Cu_3_Ti_4_O_12_ Ceramics Prepared by Mechanical Ball Milling and Low Temperature Conventional Sintering

**DOI:** 10.3390/ma15093173

**Published:** 2022-04-27

**Authors:** Mohamad M. Ahmad, Adil Alshoaibi, Sajid Ali Ansari, Tarek S. Kayed, Hassan A. Khater, Hicham Mahfoz Kotb

**Affiliations:** 1 Department of Physics, College of Science, King Faisal University, P.O. Box 400, Al-Ahsa 31982, Saudi Arabia; adshoaibi@kfu.edu.sa (A.A.); sansari@kfu.edu.sa (S.A.A.); hkhater@kfu.edu.sa (H.A.K.); 2 Department of Physics, Faculty of Science, The New Valley University, El-Kharga 72511, Egypt; 3 Department of Basic Engineering Sciences, College of Engineering, Imam Abdulrahman Bin Faisal University, Dammam 34221, Saudi Arabia; tkayed@iau.edu.sa; 4 Physics Department, Faculty of Science, Assiut University, Assiut 71516, Egypt

**Keywords:** ceramics, sintering, relative permittivity, relaxation

## Abstract

In the current study, Bi_2/3_Cu_3_Ti_4_O_12_ (BCTO) ceramics were prepared by mechanical ball mill of the elemental oxides followed by conventional sintering of the powder without any pre-sintering heat treatments. The sintering temperature was in the range 950–990 °C, which is 100–150 °C lower than the previous conventional sintering studies on BCTO ceramics. All the ceramic samples showed body-centered cubic phase and grain size ≈ 2–6 μm. Sintering temperature in the range 950–975 °C resulted in comparatively lower dielectric loss and lower thermal coefficient of permittivity in the temperature range from −50 to 120 °C. All the BCTO ceramics showed reasonably high relative permittivity. The behavior of BCTO ceramics was correlated with the change in oxygen content in the samples with sintering temperature. This interpretation was supported by the measurements of the energy dispersive x-ray spectroscopy (EDS) elemental analysis and activation energy for conduction and for relaxation in the ceramics.

## 1. Introduction

Materials with giant relative permittivity (ε′) and low dielectric loss tan δ (=ε″/ε′, where ε′ and ε″ are the real and imaginary part of the complex relative permittivity) are attractive as they can be used in multilayer capacitors and reduce the size of electronic components. In this regard, the family ACu_3_Ti_4_O_12_ (ACTO) (e.g., A = Ca, Sr, Cd, Bi_2/3_, or Ln_2/3_; Ln is a lanthanide element) drew the attention of researchers since the work of Subramanian et al. [1] in 2000. The data in ref. [1] revealed the interesting dielectric properties of ACTO ceramics in terms of giant relative permittivity which is stable over a wide range of frequencies and temperatures. CaCu_3_Ti_4_O_12_ (CCTO) has been the most studied member of the ACTO family [2,3,4,5,6,7,8]. The origin of the giant relative permittivity in ACTO ceramics (non-ferroelectric) is commonly explained in terms of the internal barrier layer capacitance (IBLC) model [9,10]. The IBLC model is applicable to the electrically heterogeneous ceramics which are composed of a semiconducting element (grain) separated by a highly resistive element (grain boundary). Therefore, under the influence of the alternating electric field, free charge carriers accumulate at the grain boundary forming internal capacitors. These capacitors result in the observed giant relative permittivity in the comparatively low frequency range (<10^5^ Hz). Bi_2/3_Cu_3_Ti_4_O_12_ (BCTO) has been reported as a promising high relative permittivity material [11,12,13,14,15,16,17,18]. In the work of Liu et al. [11], BCTO ceramics were prepared by the standard solid state reaction (SSR) technique. The process included calcination in air at 950–1000 °C for 20 h with intermediate grinding followed by sintering at 1100 °C for 10 h. The sintered BCTO ceramics showed high relative permittivity (ε′~1800 at 12.6 kHz and 300 K) that is stable over a wide range of frequencies and temperatures. Using a comparatively low temperature semi-wet route, BCTO ceramic shows ε′ of 3.6 × 10^3^ and high tan δ of 0.25 at 1 kHz [14]. BCTO was also successfully prepared by powder synthesis using sol-gel technique followed by calcination 800 °C for 10 h and sintered at 1000 °C for 20 h. This method has resulted in ε′ of 1.1 × 10^4^ and tan δ of 0.11 at 1 kHz [16]. In the same study, BCTO ceramics were also prepared by the standard SSR technique and showed lower performance (ε′~3.2 × 10^3^ and tan δ~0.11 at 1 kHz). Jesus et al. studied the effect of laser and conventional sintering (960–1000 °C, 2 h) on the dielectric properties of BCTO powder prepared by modified polymeric precursor route [17]. Conventionally sintered ceramics showed ε′ > 10^3^ but high dielectric loss (tan δ > 0.1 at 100 kHz). Laser-sintered BCTO ceramic revealed a degraded relative permittivity (ε′ < 10^2^) which was correlated to the high resistivity of grains. In the current study, we investigated the dielectric properties of BCTO, prepared by a comparatively low temperature process based on high energy mechanical milling of the elemental oxides, followed by conventional sintering without any prior calcination step. This process hereinafter is referred to as reactive sintering. We demonstrated that the phase formation starts during the milling stage and continues during the subsequent sintering step. We used sintering temperatures in the range 950–990 °C to avoid melting of the sample. Therefore, the proposed process is shorter in time and takes place at a lower temperature compared to the standard SSR.

## 2. Experiment Procedure

### 2.1. Materials

High-purity Bi_2_O_3_ (99.9%, Aldrich, Saint Louis, MO, USA), CuO (99.995%, Aldrich), and TiO_2_ (99.9%, Aldrich) were used for the synthesis of the powder of Bi_2/3_Cu_3_Ti_4_O_12_.

### 2.2. Synthesis of Bi_2/3_Cu_3_Ti_4_O_12_ Ceramics

Stoichiometric amounts of the elemental oxides were milled in Fritsch P-7 premium line ball mill using pot and balls of tungsten carbide with the balls to powder mass ratio being 10:1. The milling process was carried out without any liquid medium for 20 h with a rotation speed of 500 rpm. Afterwards, the prepared powder was collected and a suitable amount was isostatically pressed into pellets of 13 mm in diameter and 1.5 mm in thickness using a uniaxial pressure of 373 MPa. The green pellets were sintered in air for 10 h inside electrical tubular furnace at the temperatures 950 °C, 975 °C, and 990 °C using a heating/cooling rate of 4 °C/min. These samples are termed BCT-950, BCT-975, and BCT-990. The chemical reaction equation is as follows:

(1) $$\frac{1}{3}{\mathrm{Bi}}_{2}O_{3}{+3\mathrm{CuO}+4\mathrm{TiO}}_{2}\;\to \;{\mathrm{Bi}}_{\frac{2}{3}}{\mathrm{Cu}}_{3}{\mathrm{Ti}}_{4}O_{12}$$

### 2.3. Characterization Methods

The morphology and elemental composition of the ceramics was examined by using a field emission scanning electron microscope (FE-SEM, Joel, SM7600F, Tokyo, Japan) and an attached energy-dispersive X-ray spectroscope (EDS) system (Inca Oxford, High Wycombe, UK). The acceleration voltage was 20 kV for SEM and EDS observations. A Bruker D8 Advance X-ray powder diffractometer (CuKα-radiation) was used for the XRD measurements in the range 20° ≤ 2θ ≤ 90° with a scan step size of 0.02°. Impedance spectroscopy (IS) measurements were performed in the temperature range 120–500 K over the frequency range 1–40 MHz at an oscillation voltage of 0.5 V. All IS measurements were performed under flow of dry nitrogen atmosphere using a turnkey concept 50 system from Novocontrol. The temperature was controlled by a Quatro Cryosystem.

## 3. Results and Discussion

**Figure 1:**
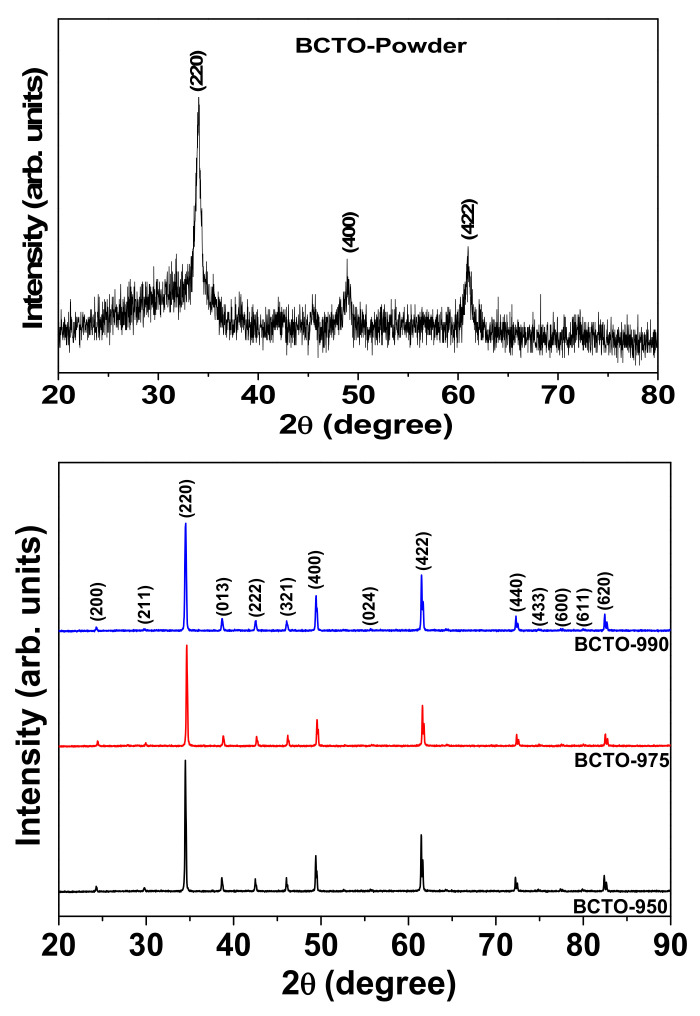
The XRD patterns of the as-synthesized powder (**top**) and the sintered ceramics (**bottom**) of BCTO.

**Figure 2:**
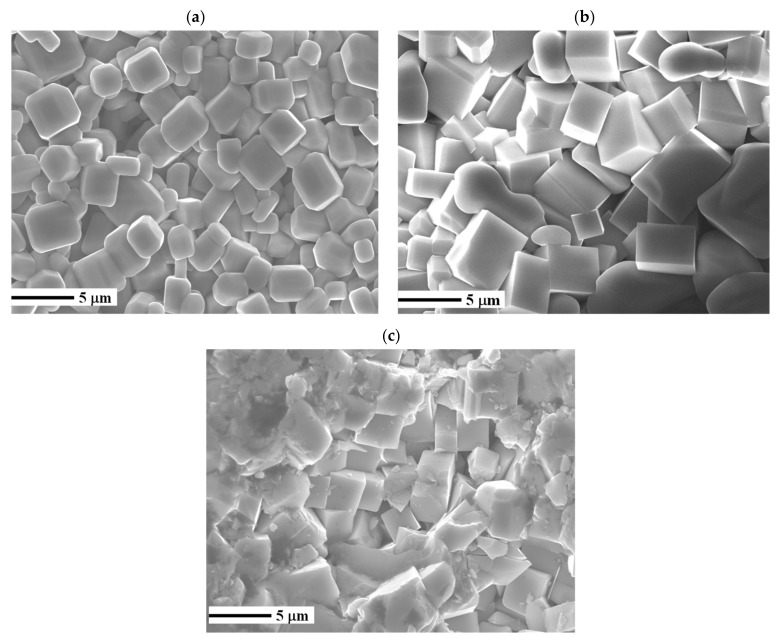
FE-SEM morphology of BCTO ceramics (**a**) BCTO-950, (**b**) BCTO-975, and (**c**) BCTO-990.

The XRD patterns of the as-synthesized BCTO powder and the sintered ceramics are demonstrated in Figure 1. It is clear that the crystalline phase has started to form in the powder during the ball mill then continued to grow during the sintering stage. The diffraction peaks of each pattern were indexed according to the body-centered cubic phase of CCTO (JCPDS card: 75–2188). The lattice parameter was calculated from the angle and hkl values of main diffraction peaks using UNITCELL software. It was found to be 7.420(4) Å, 7.421(6) Å, and 7.416(3) Å for BCTO-950, BCTO-975, and BCTO-990, respectively, which agrees with the reported literature values for BCTO [11,14]. These results suggest that the used reactive sintering method is successful to prepare the BCTO ceramics. As seen in the SEM micrographs of Figure 2, all the ceramic samples show uniformly distributed grains. Using the linear intercept method, the average grain size (D) is calculated as D = 1.56 L/MN, where L is the random line length on the micrograph, M is the magnification of the micrograph, and N is the number of the grain boundaries [19]. The calculated D values are ~2.9 ± 0.8 μm, 5.1 ± 0.9 μm, and 3.9 ± 0.3 μm for the ceramic samples BCT-950, BCT-975, and BCT-990, respectively, which is comparable to the literature values [10,15]. The relative density of BCTO ceramics, measured from the mass and geometrical dimensions of the pellet, was found to be ~95.3%, 96.2%, and 96.8% with increasing the sintering temperature.

**Figure 3:**
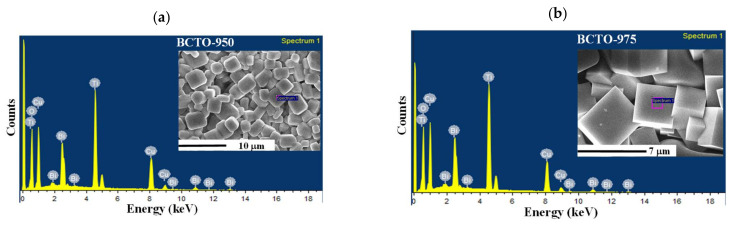

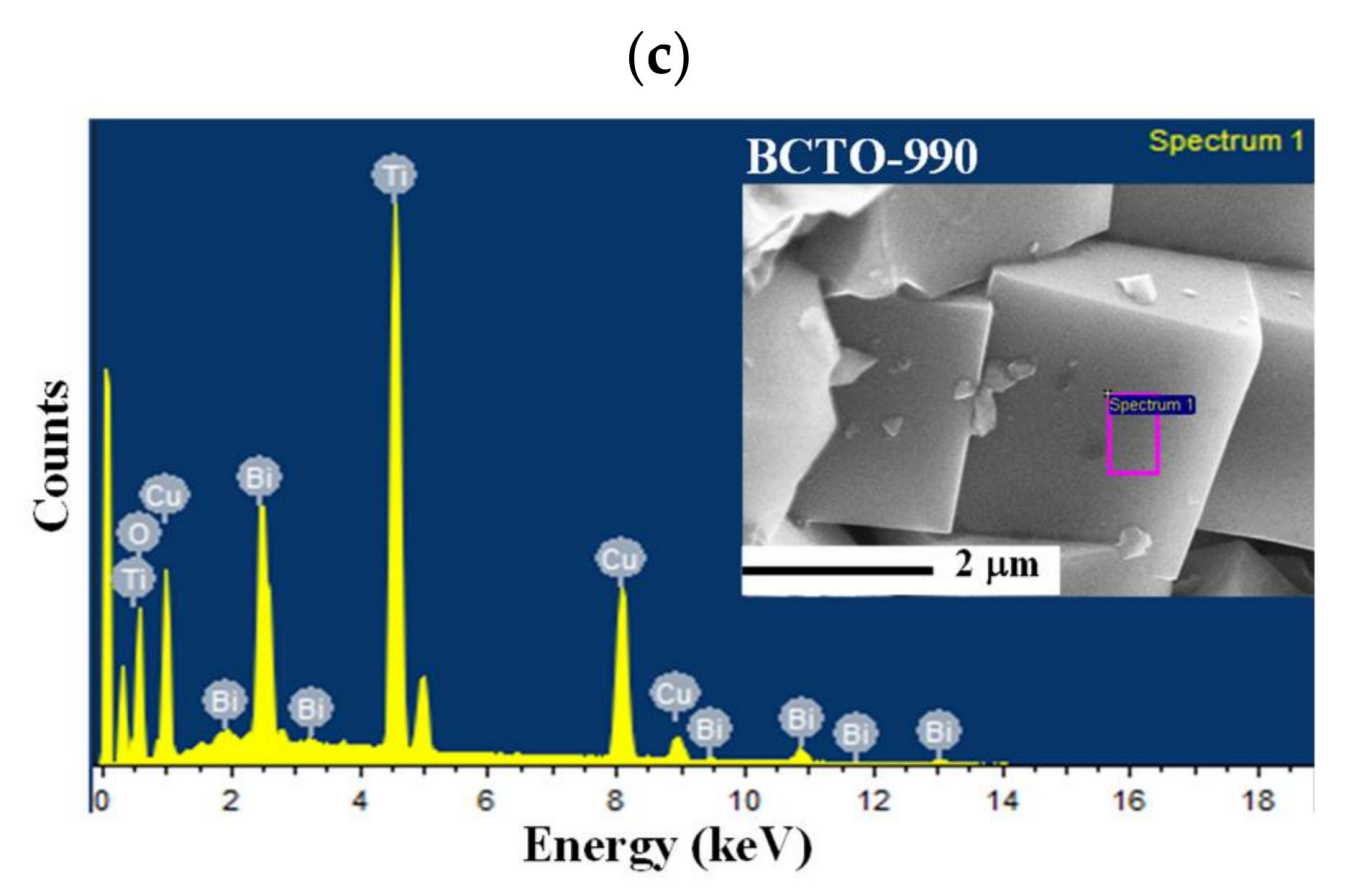
EDS spectra of (**a**) BCTO-950, (**b**) BCTO-975, and (**c**) BCTO-990 ceramic samples.

Figure 3 shows the EDS spectra for the BCTO ceramics. The presence of Bi, Cu, Ti, and O in the ceramics was confirmed and their atomic percentage is given in Table 1. As seen from the table, the ratio between elements corresponds to the nominal values for BCTO composition. Moreover, the oxygen content in the ceramic samples BCTO-950 and BCTO-975 is higher than in the ceramic BCTO-990.

**Figure 4:**
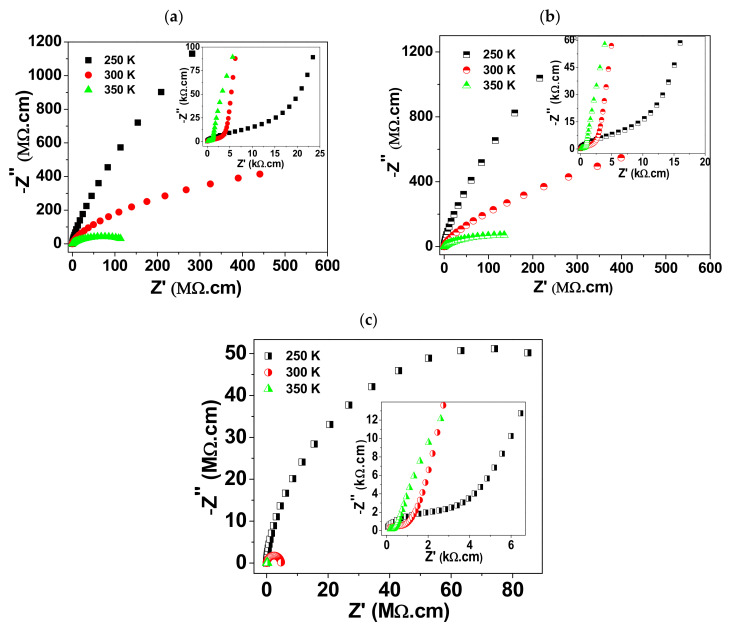
–Z′′ versus Z′ plots at selected temperatures for (**a**) BCTO-950, (**b**) BCTO-975, and (**c**) BCTO-990 ceramic samples. Insets show the expanded view of the high frequency region (close to the origin) for each sample.

Figure 4a–c depicts the complex impedance plots for the current BCTO ceramics at selected temperatures. Two semicircular arcs are recognizable on the plot of the complex impedance at a given temperature. The resistance of each element can be calculated from the intercept of the corresponding arc with the horizontal axis [20]. The calculated room temperature resistivities of the ceramics are given in Table 2. As seen in the insets of the Figure 4a–c, the arc at high frequency (close to the origin) would be correlated with an element (grain) that is electrically less resistive than that element related to the low frequency arc (grain boundary). Therefore, these arcs highlight the electrical heterogeneity of the ceramics. For all the samples, the resistivity of grain boundary is several orders of magnitude higher than that of the grain. Moreover, the samples BCTO-950 and BCTO-975 showed higher resistance than the BCTO-990. According to the results of the EDS, the samples BCTO-950 and BCTO-975 have higher oxygen content than BCTO-990. The oxygen content in CCTO-derived materials plays an important role in controlling the density of free electrons. The latter is a determinant factor for the resistance of the material. This can be described by the following equations, which are written according to Kroger–Vink notation:

(2) $$O_{O}\;\Leftrightarrow \;V_{O}{+1/2O}_{2}$$

(3) $$V_{O}\;\Leftrightarrow \;V_{O}^{•}+e’$$

(4) $$V_{O}^{•}\;\Leftrightarrow \;V_{O}^{••}+e’$$

where $V_{O}^{•}$ and $V_{O}^{••}$ represent single and double ionized oxygen vacancies.

It is worth noting that a re-oxidation was suggested to take place favorably at the grain-boundary during the cooling of the ceramic, after sintering at high temperatures. This process then contributes to the resistivity difference between grain and grain boundary in the sintered ceramics. Moreover, as seen in Table 2, the electrical properties of the grain-boundary are more sensitive to the sintering temperature than grains.

**Figure 5:**
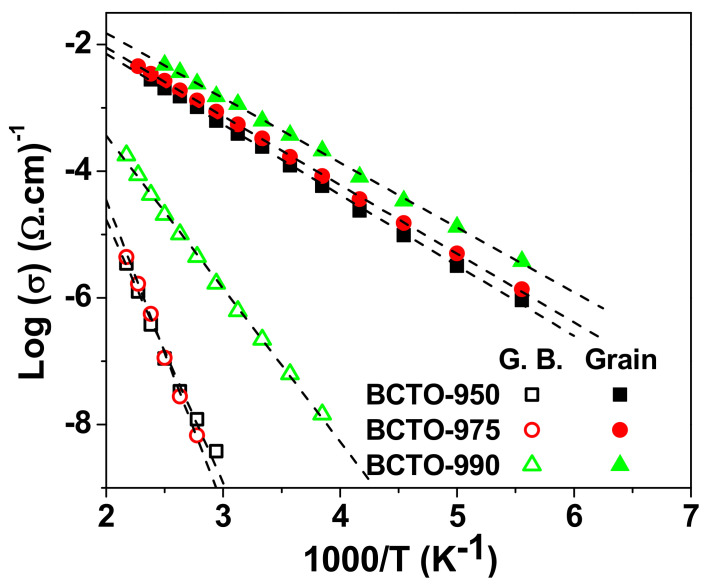
Arrhenius plots of the dc conductivity of grain (G.) and grain boundary (G.B.) for BCTO ceramics.

Figure 5 shows the Arrhenius plots of the grain and grain boundary conductivities for the investigated BCTO ceramics. The activation energy (E) for conduction was calculated from the Arrhenius relationship:

(5) $$\sigma_{G.,\;G.B.}=\;\sigma_{0\;}e^{-\left(\frac{E_{G,\;G.B.}}{K_{B}T}\right)}$$

where σ_0_ is the pre-exponential factor, K_B_ is Boltzmann constant, and T is absolute temperature. The determined values of E are given in Table 2. The activation energy for conduction in the grain and grain boundary decrease from 0.231 eV to 0.197 eV and from 0.848 eV to 0.513 eV, respectively, with increasing sintering temperature.

**Figure 6:**
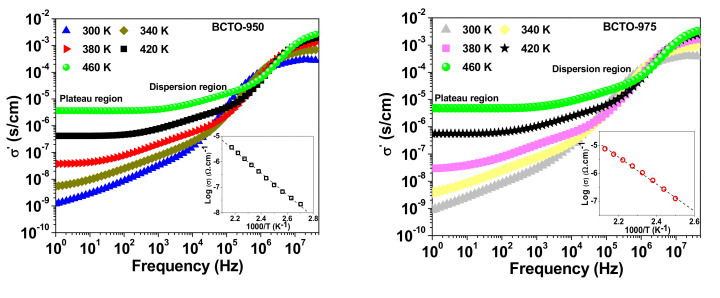

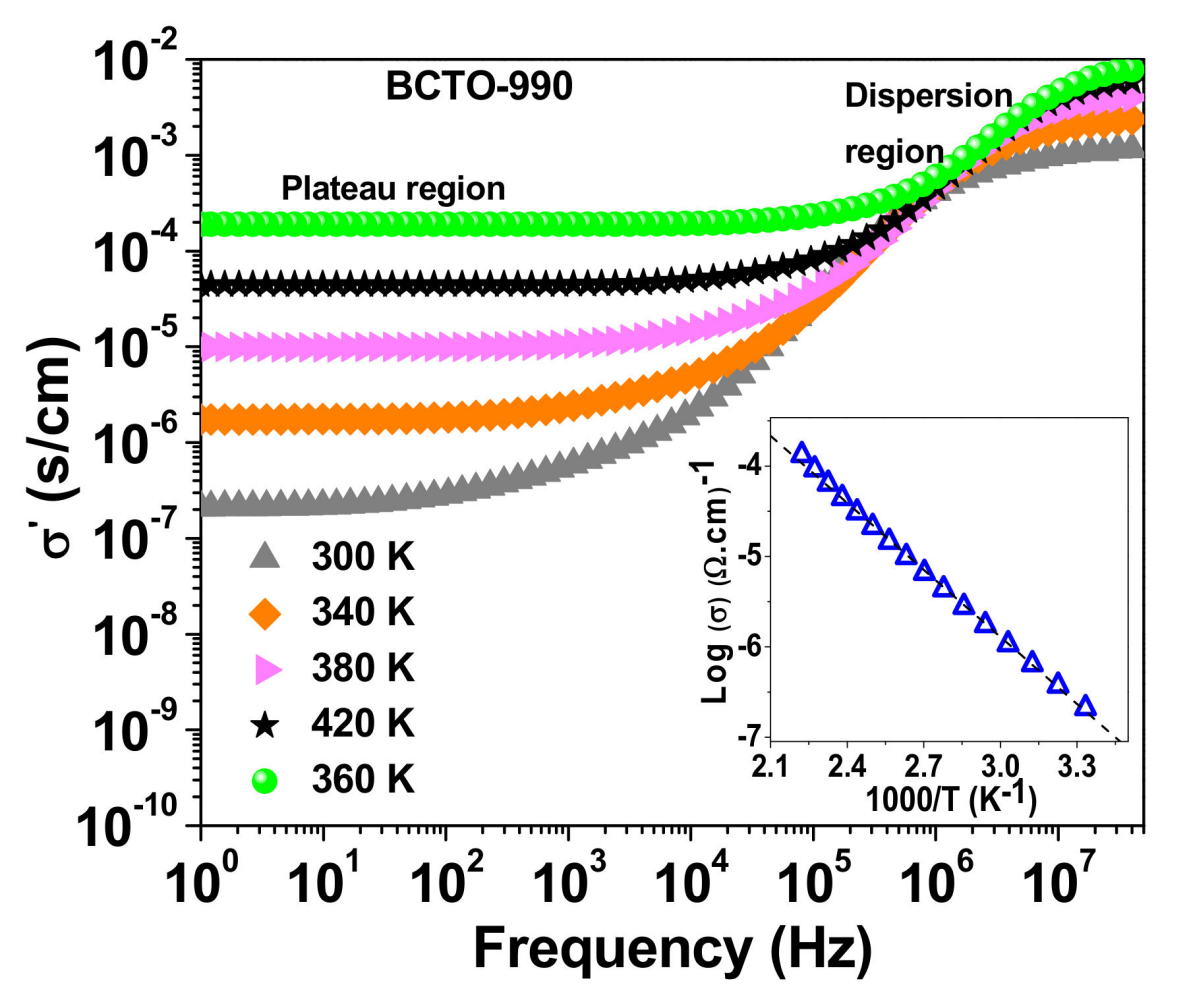
Spectra of AC conductivity at selected temperatures for BCTO ceramics. The inset in each figure shows the Arrhenius plot of DC conductivity. The dotted line represents the line of best fit.

Figure 6 shows the frequency dependence of the real part (σ′) of the complex AC conductivity of the BCTO ceramics at selected temperatures. It is noticed that a step-like decrease (dispersion region) in σ′ takes place with decreasing frequency due to the blocking effect of grain boundary. Moreover, σ′ tends to be frequency independent (plateau region) at very low frequency and at high temperatures. The conductivity at the plateau region, to a good approximation, corresponds to the DC conductivity (σ_dc_) of the sample. The insets of Figure 6 depict the Arrhenius plots for the DC conductivity according to the relationship $\sigma_{\mathrm{dc}}=\;\sigma_{0\;}e^{-\left(\frac{E_{\mathrm{dc}}}{K_{B}T}\right)}$, where σ_0_ is the pre-exponential factor and E_dc_ is the activation energy for dc conduction. The calculated values for E_dc_ are 0.848 eV, 0.887 eV, and 0.493 eV for the ceramics BCTO-950, BCTO-975, and BCTO-990, respectively. It is noticed that E_dc_ values are very close to the E_G.B_, indicating that the dc conduction process is most related to the electric response of grain boundaries for the BCTO ceramics.

**Figure 7:**
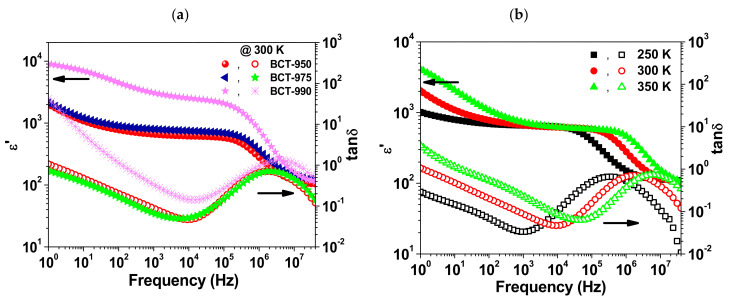

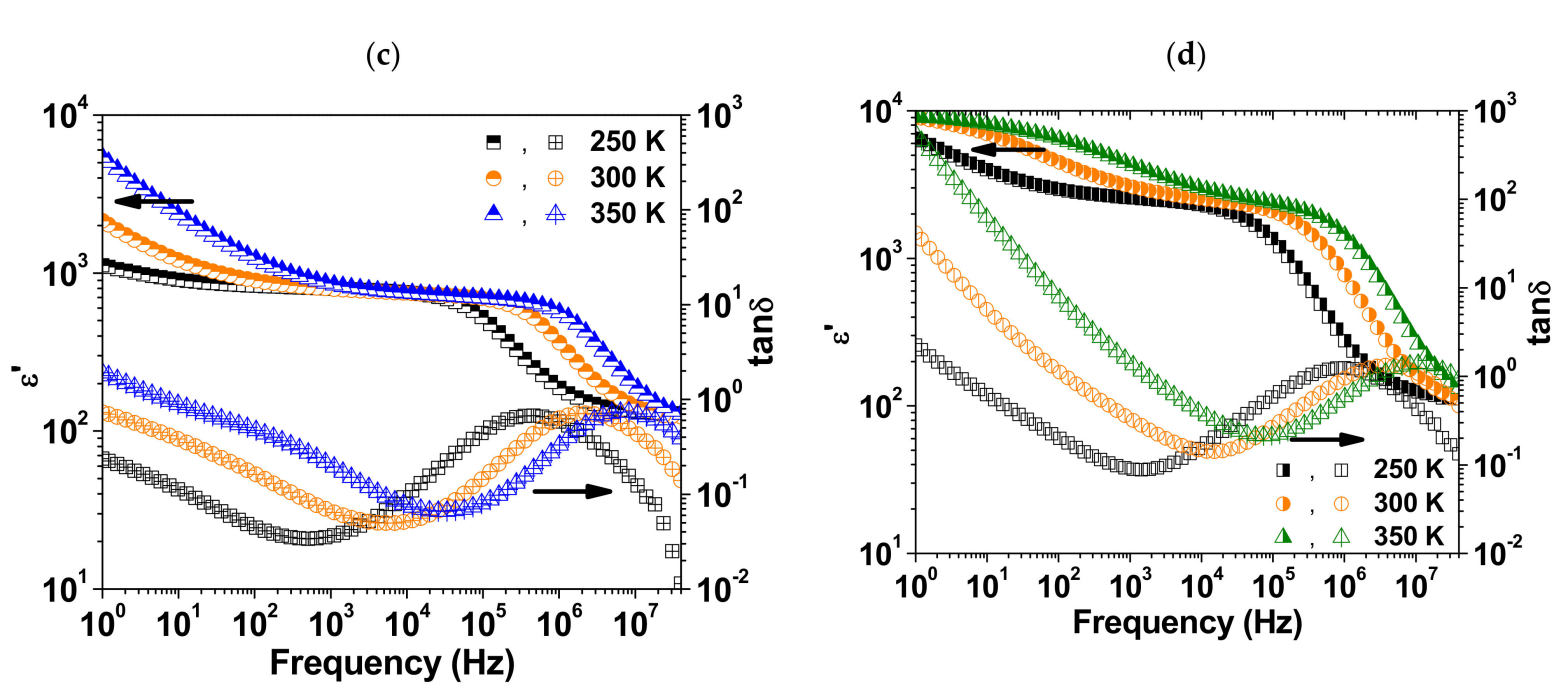
Room temperature spectra of ε′ and tan δ for all BCTO ceramics (**a**); the evolution of ε′ and tan δ spectra with increasing measuring temperature for (**b**) BCTO-950, (**c**) BCTO-975, and (**d**) BCTO-990 ceramic samples.

Figure 7 shows the room temperature spectra of ε′ and tan δ for the BCTO ceramics. The values of ε′ and tan δ at 1.1 kHz as well as the minimum value of tan δ for each sample are summarized in Table 3. On one hand, the sample BCTO-990 showed ε (~3.04 × 10^3^) that is larger than BCTO-950 (~659) and BCTO-975 (~792). On the other hand, the sample BCTO-950 exhibited the lowest tan δ of 0.04 at 7 kHz. Moreover, at a given temperature, all the samples showed a low frequency plateau region in the spectra of ε′ followed by a step-like decrease with increasing the frequency. This step is accompanied by a peak in the spectra of tan δ. With increasing temperature, the peak of tan δ shifted towards higher frequencies which indicated a thermally activated process. In general, the frequency and temperature-dependent dielectric behavior of the investigated BCTO ceramics is quite similar to what was reported previously for BCTO ceramics prepared by other methods [11,12,13,14]. The relative permittivity and dielectric loss of the current samples indicate similar performance as for SSR BCTO ceramics [11]. Higher relative permittivity (ε′~1.1 × 10^4^ at 1 kHz) was reported for BCTO ceramics prepared by a sol-gel-based method [16]. Nevertheless, these ceramics showed higher dielectric loss (tan δ~0.11 at 1 kHz) than the SSR BCTO ceramics (tan δ~0.04) of the same study.

**Figure 8:**
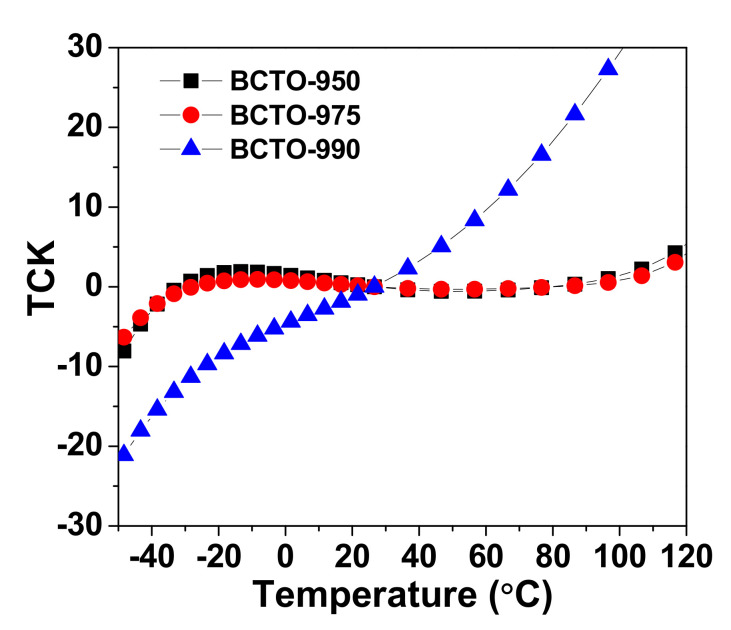
Temperature dependency of the thermal coefficient of relative permittivity (TCK) for the BCTO ceramics.

The thermal coefficient of relative permittivity (TCK) is an important factor that is related to the temperature stability of relative permittivity. TCK is given as [21,22]:

(6) $$\mathrm{TCK}=\;\left(\frac{\Delta \varepsilon_{T}^{’}}{\varepsilon_{\mathrm{RT}}^{’}}\right)=\;\left(\frac{\varepsilon_{T}^{’}-\varepsilon_{\mathrm{RT}}^{’}}{\varepsilon_{\mathrm{RT}}^{’}}\right)\times 100$$

where $\varepsilon_{T}^{’}$ is the relative permittivity at the temperature T and $\varepsilon_{RT}^{’}$ is relative permittivity at room temperature. Figure 8 shows the temperature dependence of TCK at 11.7 kHz for BCTO ceramics. Interestingly, the TCK values of the ceramics BCTO-950 and BCTO-975 were −8 to +4% in the temperature range −50–120 °C. Therefore, the BCTO ceramics prepared by reactive sintering at comparatively lower temperatures satisfied the Electronic Industry Association standard (EIA) X7R capacitor requirements (TCK < ±15% in the temperature interval −55–125 °C) [23,24].

**Figure 9:**
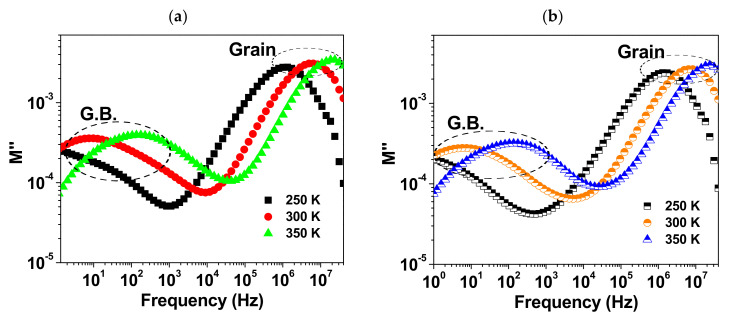

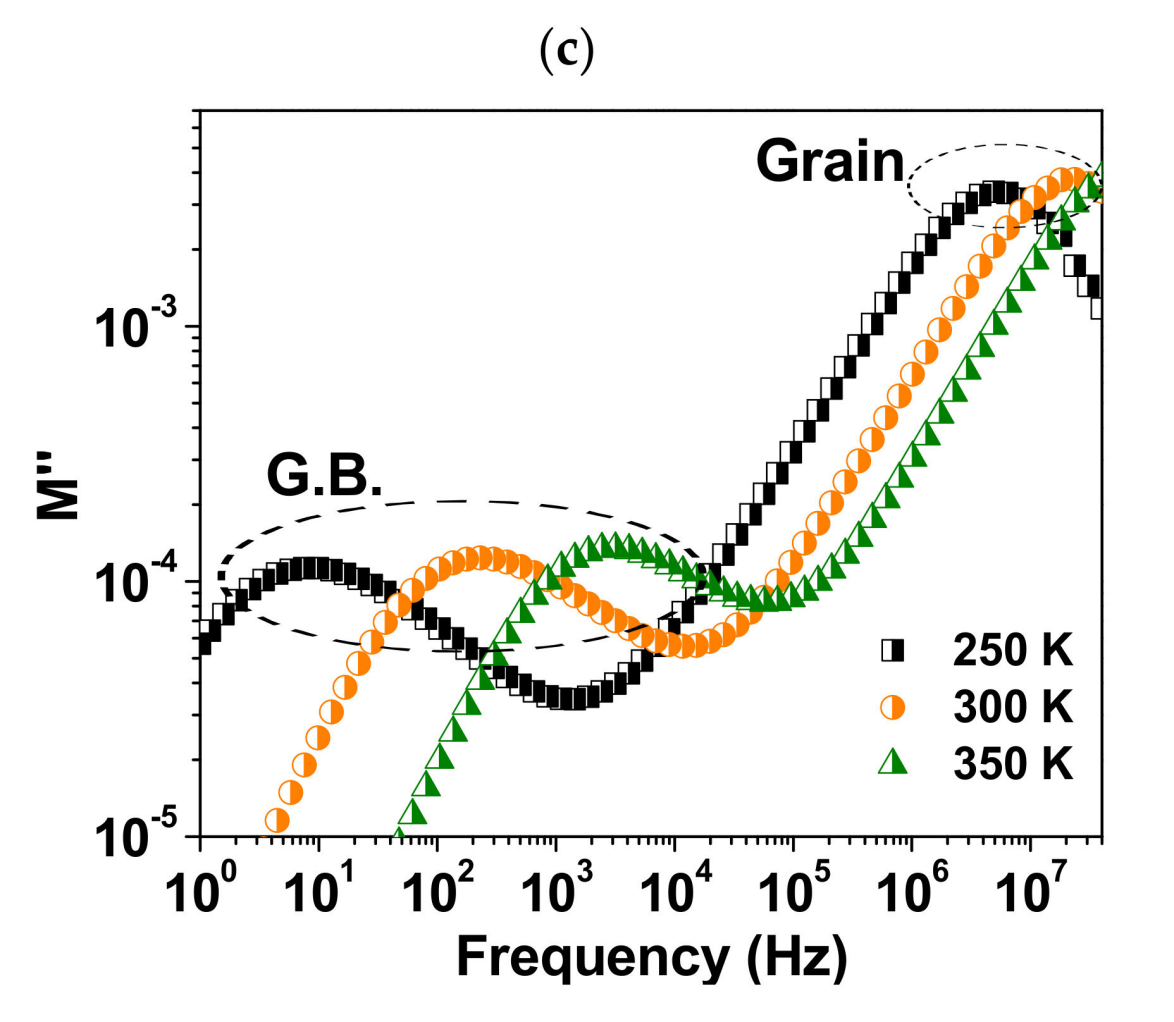
Frequency dependence of M″ at different temperatures for (**a**) BCTO-950, (**b**) BCTO-975, and (**c**) BCTO-990 ceramic samples.

The spectra of the imaginary part of modulus (M″) at selected temperatures are shown in Figure 9a–c. Two relaxation peaks are observed in each spectrum. It is known that the peak maximum of M″ ($M_{\max}^{}$) is inversely proportional to the capacitance (C) of the element responsible for the relaxation peak, i.e., $M_{\max}^{}\;$= C_0_/2C, where C_0_ is the empty cell capacitance [25,26]. Therefore, the low and high frequency relaxation peaks of M″ could be attributed to the response of grain-boundary and grain contributions, respectively, as the grain-boundary has higher resistance and capacitance compared to the grain [11,25]. The calculated values for the capacitance of grain (C_G._) and grain boundary (C_G.B._) are given in Table 2. It is observed that the grain boundary capacitance is around ten times larger than that of grain for BCTO-950 and BCTO-975, which is similar to the results in Refs [11,12]. The ratio C_G.B._/C_G._ for BCTO-990 is found to be ~30 which is correlated with its comparatively higher relative permittivity. Moreover, as seen in Figure 9, both of the relaxation peaks in M″ spectra are found to shift towards higher frequency with increasing the temperature which indicates thermally active processes. The mean value of the relaxation time (τ) is related to the frequency corresponding to the peak maximum of M″ (f_max_) as τ= 1/2πf_max_. The dependence of τ on the inverse of temperature was found to follow the Arrhenius law:

(7) $$\tau =\;\tau_{0\;}e^{-\left(\frac{E_{R}}{k_{B}T}\right)}$$

where τ_0_ is the prefactor and E_R_ is the activation energy for the relaxation process.

**Figure 10:**
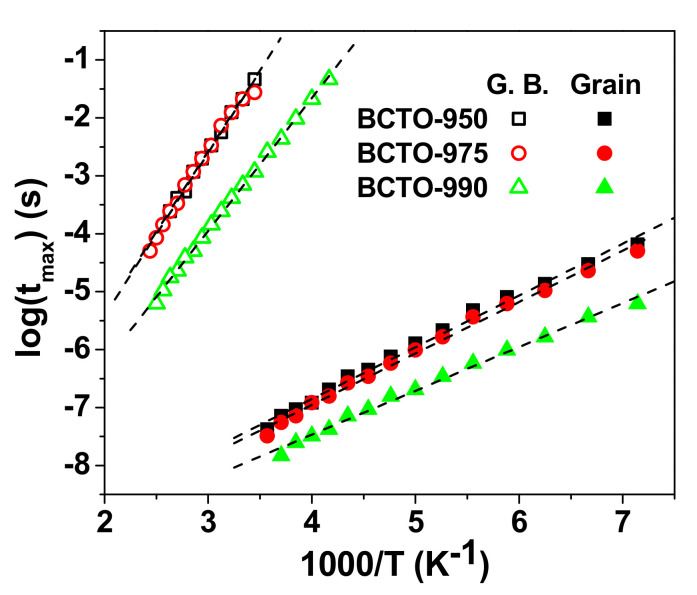
The Arrhenius plots of the relaxation time τ in grain and grain boundary for BCTO ceramics. The dashed line represents the line of best fit.

The Arrhenius plots of the relaxation time τ in grain and grain boundary for BCTO ceramics are given in Figure 10. The calculated values of E_R_ in grain and grain boundaries are included in Table 2. These values are close to the reported values for BCTO ceramics [11,13].

The activation energy values of Table 2 are close to the reported activation energy for oxygen vacancies in titanate-based materials [27]. Therefore the dielectric behavior of the current BCTO ceramics is correlated to the oxygen vacancy content which developed during sintering.

## 4. Conclusions

In this investigation, Bi_2/3_Cu_3_Ti_4_O_12_ (BCTO) ceramics were successfully prepared by mechanical ball mill of the powder followed by conventional sintering at comparatively low temperatures (950–990 °C). The XRD measurements confirmed the body-centered cubic perovskite-related structure for BCTO ceramics. The average grain size increased slightly with increase the sintering temperature. At room temperature, BCTO ceramics showed high relative permittivity (>660) over a wide range of frequencies (1–10^5^ Hz). Nevertheless, the dielectric loss showed considerable dependence on the sintering temperature. The ceramics sintered at (950–975 °C) showed low thermal coefficient of relative permittivity of ~−8 to +4% in the temperature range −50–120 °C, and minimum low dielectric loss of ~0.04–0.05. The calculated values for the activation energy for conduction and for relaxation process were found close to the reported activation energy for oxygen vacancies in titanate based materials. The variation in the properties of the current BCTO ceramics was correlated with the change in oxygen content in the ceramics with sintering temperature.

## Figures and Tables

**Table 1:** EDS quantitative elemental analysis for BCTO ceramic samples.

	Atomic Percentage (at %)				Bi:Cu:Ti:O
	Bi	Cu	Ti	O	
BCTO-950	2.58 ± 0.02	11.81 ± 0.03	14.83 ± 0.11	70.78 ± 0.12	1:4.6:5.8:27.4
BCTO-975	2.55 ± 0.02	11.58 ± 0.02	15.66 ± 0.13	70.21 ± 0.13	1:4.5:6.1:27.5
BCTO-990	3.57 ± 0.01	16.87 ± 0.06	21.83 ± 0.15	57.52 ± 0.20	1:4.5:6.1:16.1

**Table 2:** Activation energy values for conduction (E) and for relaxation process (E_R_) in grain (G.) and grain boundary (G.B.); the resistivity of grain (R_G._) and grain boundary (R_G.B._); and the capacitance of grain (C_G._) and grain boundary (C_G.B._) for BCTO ceramics.

	E_(G.)_ (eV)	E_(G.B.)_ (eV)	E_R(G.)_ (eV)	E_R(G.B.)_ (eV)	R_G._ (Ω·cm)	C_G._ (F)	R_G.B._ (Ω·cm)	C_G.B._ (F)
BCTO-CS950	0.231	0.848	0.176	0.557	4134	8.3 × 10^−11^	>10^9^	7.2 × 10^−10^
BCTO-CS975	0.214	0.931	0.176	0.553	3038	8.2 × 10^−11^	>10^9^	9.3 × 10^−10^
BCTO-CS990	0.197	0.513	0.150	0.454	1600	10.4 × 10^−11^	4.6 × 10^6^	3.1 × 10^−9^

**Table 3:** The room temperature values of ε′ and tan δ at 1.1 kHz and the minimum dielectric loss value tan δ)_min_ for BCT ceramics.

	Ε′	tan δ	tan δ)_min_
	at 1.1 kHz		
BCT-CS950	660	0.06	0.04 (at 7 kHz)
BCT-CS975	792	0.08	0.05 (at 9 kHz)
BCT-CS990	3040	0.32	0.14 (at 15 kHz)

## Data Availability

Not applicable.
